# Relation of Serum Leptin and Adiponectin Level to Serum C-Reactive Protein: The INTERLIPID Study

**DOI:** 10.1155/2013/601364

**Published:** 2013-11-24

**Authors:** Yasuyuki Nakamura, Hirotsugu Ueshima, Nagako Okuda, Katsuyuki Miura, Yoshikuni Kita, Tomonori Okamura, Akira Okayama, Sohel R. Choudhury, Beatriz Rodriguez, Kamal H. Masaki, Jeremiah Stamler

**Affiliations:** ^1^Cardiovascular Epidemiology, Kyoto Women's University, Kyoto 605-8501, Japan; ^2^Department of Health Science, Shiga University of Medical Science, Otsu 520-2121, Japan; ^3^Department of Nutritional Epidemiology, National Institute of Health and Nutrition, Tokyo 162-8636, Japan; ^4^Department of Preventive Medicine and Public Health, Keio University, Tokyo 160-8582, Japan; ^5^First Institute for Health Promotion and Health Care, Tokyo 101-0061, Japan; ^6^Department of Epidemiology and Research, National Heart Foundation Hospital & Research Institute, Dhaka 1216, Bangladesh; ^7^John A. Burns School of Medicine, University of Hawaii, Honolulu, HI 96813, USA; ^8^Feinberg School of Medicine, Northwestern University, Chicago, IL 60611, USA

## Abstract

*Objective*. Despite considerable study, the relevance of leptin and adiponectin for atherosclerosis development is still unsettled. We investigated relations of serum leptin and adiponectin to serum C-reactive protein (CRP), using the INTERLIPID dataset on Japanese emigrants living in Hawaii and Japanese in Japan. *Design and Methods*. Serum leptin, adiponectin, and CRP were measured by standardized methods in men and women of ages 40 to 59 years from two population samples, one Japanese-American in Hawaii (83 men, 89 women) and the other Japanese in central Japan (111 men, 104 women). Participants with CRP >10 mg/L were excluded. *Results*. Sex-specific multiple linear regression analyses, with log-transformed leptin and adiponectin (log-leptin, log-adipo), site (Hawaii = 1, Japan = 0), SBP, HbA1c, smoking (cigarettes/day), and physical activity index score of the Framingham Offspring Study as covariates, showed that log-leptin directly related and log-adipo inversely related to log-CRP for both sexes (*P*s < 0.05 to <0.01). Addition to the model of BMI and interaction terms (BMI × log-leptin, BMI × log-adipo, SITE × log-leptin, SITE × log-adipo) resulted in disappearance of statistical significance except for direct relation of log-leptin to log-CRP in men (*P* = 0.006). *Conclusions*. Leptin directly related to CRP independent of BMI and other confounding factors in men but not in women.

## 1. Introduction

Recent advances have illuminated the role of inflammation and underlying cellular and molecular mechanisms in atherogenesis [1]. C-reactive protein (CRP), a marker of inflammation, has been recognized as an indicator of atherosclerotic and cardiovascular risk [2]. Leptin and adiponectin, secreted by adipose tissue, represent the most abundant adipokines in human serum [3–7]. Recent studies have implicated leptin as a risk factor for cardiovascular diseases independent of traditional risk factors [5]. Adiponectin, on the other hand, may have anti-inflammatory, antiatherogenic, and antidiabetic properties [8, 9]. Lower serum adiponectin concentrations are reported to be associated with coronary heart disease (CHD) risk [10]. As to the relationship between these adipocytokines and CRP direct association of leptin with CRP, and inverse association of adiponectin with CRP have been reported [11–14]. Adjustment for BMI or other obesity measures was done in these previous studies; interaction terms between adipocytokines and obesity measure were not reported.

 INTERLIPID, an ancillary study of the International Study of Macro/micronutrients and Blood Pressure (INTERMAP), investigated CHD risk factors in four Japanese population samples in Japan and a Japanese-American population sample in Hawaii [15–17]. In INTERMAP, dietary surveys were conducted with a highly standardized protocol in 17 random population samples in four countries (Japan, China, UK, and USA) [18, 19]. 

 Our data, from Japanese in Japan and Japanese-Americans, that is, an ethnically homogenous cohort with a wide range of BMIs (from 17.6 to 47.0 kg/m^2^), enable us to address unsolved problems on relationships between leptin, adiponectin, and CRP, including interaction terms.

## 2. Methods 

### 2.1. Participants

Detailed methods of the INTERMAP Study have been described [18, 19]. INTERLIPID participants of ages 40–59 years were from five INTERMAP population samples: four in Japan and one in Hawaii [18, 19]. For the present study, serum leptin and adiponectin concentrations were measured in individuals from two of these samples, one from Japan and one from Hawaii. The two population samples were (1) Japanese residents in Aito town, a rural town in Shiga prefecture, central Japan (129 men and 129 women) and (2) third and fourth generation offspring of Japanese emigrants living in Honolulu, Hawaii (100 men and 106 women). Participants in Honolulu were asked about the ethnicity of their mother and father; those included in the study responded 100% Japanese to both parents. Among those in these two samples, 65 persons (34 Japanese, 31 Japanese-Americans) were excluded because volume of their stored serum specimen was not enough to measure CRP, adiponectin, and leptin; 12 persons (9 Japanese, 3 Japanese-American) were excluded because their serum CRP concentrations were more than 10 mg/L, leaving 215 Japanese individuals (111 men and 104 women) and 172 Hawaiian Japanese Americans (83 men and 89 women). 

 Ethics committees of the Shiga University of Medical Science, the Pacific Health Research Institute, and Northwestern University approved the study protocol. Written informed consent was obtained from all participants. 

### 2.2. Anthropometric and Lifestyle Assessment

Participants visited the research centers four times on two pairs of consecutive days on average three weeks apart. Height and weight with light clothes were measured at each visit. Four measurements of height and weight were averaged. Two standardized BP measurements were made on each of four different days. Eight measurements of BP were averaged. Using a questionnaire, trained observers inquired about physical activity, smoking status, previous medical history of cerebrocardiovascular diseases/diabetes, use of medication (including antihypertensive medication), and so forth. BMI was calculated as weight divided by height squared (kg/m^2^). To evaluate physical activity, questions were posed about number of hours per day spent in heavy activity, moderate activity, light activity, watching TV, other sedentary, and no activity (sleeping); the interviewer ensured that the total time added up to 24 h. A physical activity index score was calculated by multiplying the time spent on different activities by corresponding weighting factors that parallel the increased rate of oxygen consumption associated with increasingly more intense physical activity; for this, the procedure in the Framingham Offspring Study [20] was followed.

### 2.3. Biochemical Measurements

For the INTERLIPID Study, nonfasting blood was drawn on the second day of the first two-day visit pair. Two timed 24 h urine collections were obtained for each participant. In addition, nonfasting blood was drawn from INTERLIPID participants [11–16]. We used data on analytes measured in these blood samples, as well as data from INTERMAP.

Serum and plasma were obtained by centrifugation within 30 min of blood drawing and immediately refrigerated. Within 24 hours, all specimens were frozen and stored locally at −70°C. Samples from the Hawaiian and Japanese centers were shipped to a central laboratory in Japan on dry ice. Individual samples from the two centers were randomly allocated for analysis to avoid systematic measurement bias. Serum leptin was measured by immunoassays (Millipore Corp., Billerica, MA, USA), serum adiponectin by an enzyme-linked immunosorbent assay using the ELISA kit (Otsuka Pharmaceutical Co., Ltd., Tokyo, Japan) [17], and C-reactive protein (CRP) by immunoturbidimetric assay, all three at the central laboratory.

 Postprandial stability of leptin and adiponectin has been shown in normal and obese persons, as well as in patients with type II diabetes mellitus [21–24].

### 2.4. Data Analyses

SAS version 9.2 for Windows (SAS Institute, Cary, NC) was used. Because the distributions of serum leptin, adiponectin, and CRP were positively skewed, logarithmic transformation was used to normalize them. Sex-specific participant characteristics were analyzed by quartile of serum CRP concentration. The Mantel-Haenszel chi-square statistical test for nominal variables and the “contrast” option for analysis of variance for continuous variables (including log-leptin and log-adiponectin) were done to assess whether or not there was a significant trend across quartiles of CRP concentration. Sex-specific multiple linear regression analysis with adjustment for confounders was used to examine the relationships of log-leptin, log-adiponectin with log-CRP. Model 1 included site (Hawaii = 1, Japan = 0), age, log-leptin, and log-adiponectin; Model 2, Model 1 covariates + cigarettes/day, physical activity index, systolic blood pressure, hemoglobin A1c, and BMI; Model 3, Model 2 covariates + interaction terms (log-leptin × BMI, log-adiponectin × BMI); Model 4, Model 2 covariates + interaction terms (log-leptin × site, log-adiponectin × site); and Model 5, Model 2 + interaction term (log-leptin × BMI, log-adiponectin × BMI, log-leptin × site, and log-adiponectin × site). All *P* values were two-tailed; *P* < 0.05 was considered significant.

## 3. Results

### 3.1. Descriptive Statistics

The range of BMI in these two population samples was 17.6 to 47.0 kg/m^2^. Characteristics of participants by quartile of serum CRP concentration for men are shown in Table 1. Mean age, hemoglobin A1c, BMI, and median leptin were significantly greater in the higher CRP concentration groups (*P*s 0.020 to <0.001). Median adiponectin was significantly lower in the higher CRP concentration groups (*P* < 0.001). Percentage of participants from Hawaii, mean cigarettes per day, and systolic blood pressure were not significantly different among the groups. Physical activity index was significantly U-shaped among the groups, shown by “contrast” option for analysis of variance (“quad”) (*P* = 0.012).

Characteristics of participants by quartile of serum CRP concentration for women are shown in Table 2. Percentage of participants from Hawaii, mean systolic blood pressure, hemoglobin A1c, BMI, and median leptin were significantly greater in the higher CRP concentration groups (*P*s 0.024 to <0.001). Mean physical activity index and median adiponectin were significantly lower in the higher CRP concentration groups (*P* < 0.001). Mean age and cigarettes per day were not different among the groups.

### 3.2. Partial Correlation Coefficients among log-CRP, BMI, log-Adiponectin, and log-Leptin, Adjusted for Age and Site

Partial correlation coefficients among log-CRP, BMI, log-adiponectin, and log-leptin, adjusted for age and site, for men and women separately, are shown in Table 3. All partial correlation coefficients were significant. Partial correlation coefficients of log-CRP, BMI, log-adiponectin, and log-leptin are slightly larger for women than men.

### 3.3. Relations of Serum log-Leptin and log-Adiponectin to log-CRP

For men, in multiple linear regression models adjusted first for site and age, log-leptin was significantly directly related to, and log-adiponectin was significantly inversely related to log-CRP (Model 1, Table 4). With addition to Model 1 of cigarettes per day, physical activity index, systolic blood pressure, HbA1c, and BMI, log-adiponectin was significantly inversely related to log-CRP; relation of log-leptin to log-CRP became nonsignificant (Model 2). With further addition to Model 2 of interaction terms, log-leptin × BMI and log-adiponectin × BMI, log-leptin was significantly directly related to log-CRP; relation of log-adiponectin to log-CRP became nonsignificant (Model 3). With addition to Model 2 of interaction terms, log-leptin × site and log-adiponectin × site, log-adiponectin was significantly inversely related to log-CRP; relation of log-leptin to log-CRP became nonsignificant (Model 4). Finally, with addition to Model 2 of all interaction terms, log-leptin × BMI, log-adiponectin × BMI, log-leptin × site, and log-adiponectin x site, log-leptin was significantly directly related to log-CRP (*P* = 0.006); relation of log-adiponectin to log-CRP became nonsignificant (Model 5). Cigarettes per day were significantly directly related to log-CRP in all models (*P* < 0.05), and BMI was significantly directly related to log-CRP in Models 2, 4, and 5 (*P* = 0.035 to 0.001).

For women, in multiple linear regression models adjusted first for site and age, log-leptin was significantly directly related to and log-adiponectin was significantly inversely related to log-CRP (Model 1, Table 5). With addition to Model 1 of cigarettes per day, physical activity index, systolic blood pressure, and BMI, relations of log-leptin and log-adiponectin to log-CRP became nonsignificant (Model 2). Unlike the data for men, nonsignificant relations of log-leptin and log-adiponectin to log-CRP did not change with addition of interaction terms (Models 3, 4, and 5).

## 4. Discussion

The main findings here are that serum leptin related to CRP independent of BMI, other possible confounding factors, and their interaction terms in men, but not in women; adiponectin did not independently relate to CRP in men or women.

Previous studies on sex differences in the relationship of leptin to CRP showed that either the relationship was similar in men and women independent of obesity [11, 12] or that the independent relationship was seen only in women [25]. This is the first study to show that the association between leptin and CRP is more distinct in men than in women. A pioneer work on this matter was performed by Shamsuzzaman et al. who studied 100 healthy volunteers and found that the association between leptin and CRP was significant after adjustment for age, BMI, and other possible confounders in men and women [11]. Ble et al. reported a direct association between leptin and CRP independent of sex or BMI [12]. Abdullah et al., on the other hand, found that leptin was associated in women with CRP independent of obesity, but not in men [25]. Results of a study by Samara et al. [26] are in agreement with those of Abdullah et al. Reasons for the differences in results here and from previous studies are not clear. Difference in ethnicity cannot be ruled out, although unlikely. Several studies showed the association of adiposity or obesity with CRP was stronger in women than men [27–31]. Another study showed this association was present in women, but not in men [32]. In the present study, we found partial correlations of log-CRP and BMI to be slightly larger in women than in men. It is understandable that the association between leptin and CRP was seen only in men in the present study because a strong correlation of log-CRP with BMI in women attenuated the association when BMI was included in the model.

 CRP is synthesized by the liver, mostly under the regulation of the proinflammatory cytokines, such as interleukin (IL)-6, IL-1, and tumor necrosis factor-*α* (TNF-*α*) [33]. Leptin induces the production of these cytokines [34]. Leptin receptor, in addition, was shown to have signaling capability of IL-6-type cytokine receptors [35]. Furthermore, a direct CRP-stimulatory activation of leptin, independent of IL-6 or other proinflammatory cytokines, has been shown [12].

 We found an inverse association between adiponectin and CRP independent of BMI in men. Our findings are consistent with those of previous studies in men [13, 14, 36]. However, the association in the present study disappeared with inclusion of interaction terms (log-leptin × BMI, log-adiponectin × BMI, log-leptin × site, and log-adiponectin × site). Because interaction terms were not considered in previous studies, we cannot compare our results with those of others. We hypothesized as to the importance of interaction terms in the setting of adipocytokines that interplay tightly with volume of adipose tissue. 

 The main strengths of the present study are (1) its population-based samples; (2) standardized collection of BP and blood data; and (3) use of multiple procedures for quality control. The study was limited by its two-sample cross-sectional design. Findings may or may not be generalizable to other populations. Due to the cross-sectional nature of this study, its results must be interpreted cautiously in regard to cause-effect relationships. Regrettably, we do not have adiposity data other than BMI, such as fat mass.

 In conclusion, serum leptin directly related to CRP independent of BMI and other confounding factors in men, but not in women. 

## Figures and Tables

**Table 1:** BMI, SBP, serum leptin, adiponectin, and other characteristics by quartile of serum CRP in men.

Variable	Quartile 1	Quartile 2	Quartile 3	Quartile 4	Trend *P*
CRP range (mg/L)	0.053–0.291	0.295–0.539	0.559–1.100	1.110–7.220	
Number (*N* = 194)	49	48	49	48	
Hawaii (%)	26.5	52.0	44.9	47.9	0.071
Age (y)	49.3 ± 6.3	48.8 ± 5.2	50.9 ± 5.7	51.4 ± 5.4	0.020
Cigarettes/day	6.8 ± 11.1	7.7 ± 12.4	7.3 ± 11.5	10.5 ± 14.2	0.188
PA index	4.7 ± 4.4	2.7 ± 3.7	3.3 ± 4.1	4.4 ± 4.5	0.928
SBP (mmHg)	121 ± 14	117 ± 13	123 ± 13	123 ± 12	0.174
HbA1c (%)	4.6 ± 0.4	4.7 ± 0.5	5.0 ± 0.8	5.0 ± 1.0	0.002
BMI (kg/m^2^)	23.4 ± 3.5	25.1 ± 3.6	26.6 ± 4.0	27.2 ± 5.0	<0.001
Leptin (mcg/L)*	3.1 (2.3, 4.5)	3.7 (3.2, 5.1)	4.5 (3.4, 5.5)	4.7 (3.3, 7.1)	<0.001
Adiponectin (mg/L)*	8.4 (5.6, 12.0)	7.0 (5.2, 9.0)	6.6 (5.1, 8.4)	5.5 (4.6, 7.2)	<0.001

*Data are shown as mean ± SD or median (25 percentile, 75 percentile). PA index: physical activity index score; SBP: systolic blood pressure; HbA1c: hemoglobin A1c; BMI: body mass index; site (Hawaii = 1, Japan = 0).

**Table 2:** BMI, SBP, serum leptin, adiponectin, and other characteristics by quartile of serum CRP in women.

Variable	Quartile 1	Quartile 2	Quartile 3	Quartile 4	Trend *P*
CRP range (mg/L)	0.054–0.211	0.230–0.494	0.505–1.150	1.160–7.400	
Number (*N* = 193)	48	48	49	48	
Hawaii (%)	12.5	35.4	61.2	75.0	<0.001
Age (y)	48.4 ± 5.9	49.8 ± 5.9	50.3 ± 5.7	49.5 ± 4.7	0.274
Cigarettes/day	0.2 ± 1.0	0	0.4 ± 2.9	0.5 ± 2.9	0.324
PA index	4.8 ± 4.5	3.6 ± 3.8	2.3 ± 3.7	2.1 ± 3.2	<0.001
SBP (mmHg)	114 ± 12	114 ± 12	117 ± 14	119 ± 14	0.024
HbA1c (%)	4.4 ± 0.3	4.6 ± 0.5	4.6 ± 0.4	4.8 ± 0.9	<0.001
BMI (kg/m^2^)	21.9 ± 1.8	23.4 ± 2.4	24.8 ± 3.5	26.9 ± 5.4	<0.001
Leptin (mcg/L)*	5.5 (4.4, 8.4)	7.8 (6.2, 10.5)	11.1 (7.9, 15.3)	14.0 (8.1, 18.8)	<0.001
Adiponectin (mg/L)*	13.1 (8.9, 18.7)	10.7 (7.7, 15.1)	10.4 (7.6, 12.8)	8.5 (5.4, 10.4)	<0.001

*Data are shown as mean ± SD or median (25 percentile, 75 percentile). PA index: physical activity index score; SBP: systolic blood pressure; HbA1c: hemoglobin A1c; BMI: body mass index; site (Hawaii = 1, Japan = 0).

**Table 3:** Partial correlation coefficient matrix among log-CRP, BMI, log-adiponectin, and log-leptin, adjusted for age and site for men and women separately—INTERLIPID Study.

Variable	log-CRP	BMI	log-Adiponectin	log-Leptin
Men				
log-CRP	1.000	0.323***	−0.281***	0.226**
BMI	—	1.000	−0.330***	0.694***
log-Adiponectin	—	—	1.000	−0.289***
log-Leptin	—	—	—	1.000

Women				
log-CRP	1.000	0.413***	−0.297***	0.382***
BMI	—	1.000	−0.345***	0.675***
log-Adiponectin	—	—	1.000	−0.389***
log-Leptin	—	—	—	1.000

Partial correlation coefficients adjusted for age and site. *P < 0.05; **P < 0.01; ***P < 0.001. log-CRP: log-transformed CRP concentration; BMI: body mass index (kg/m^2^); log-adiponectin: log-transformed adiponectin concentration; log-leptin: log-transformed leptin concentration.

**Table 4:** Relations of serum log-leptin and log-adiponectin to log-CRP in men.

Variable	Model 1		Model 2		Model 3		Model 4		Model 5	
	*β*	*P *	*β*	*P *	*β*	*P *	*β*	*P *	*β*	*P *
Site	0.047	*0.505 *	0.023	*0.782 *	0.002	*0.985 *	−0.457	*0.213 *	−1.388	*0.005 *
Age	0.010	*0.066 *	0.008	*0.132 *	0.006	*0.225 *	0.008	*0.118 *	0.007	*0.198 *
log-Leptin	0.312	*0.030 *	0.110	*0.574 *	1.600	*0.013 *	0.233	*0.344 *	2.054	*0.006 *
log-Adiponectin	−0.530	*0.001 *	−0.395	*0.016 *	−0.528	*0.544 *	−0.674	*0.001 *	1.157	*0.257 *
Cigarettes/day			0.006	*0.033 *	0.007	*0.014 *	0.007	*0.019 *	0.007	*0.015 *
PA index			0.003	*0.644 *	0.006	*0.429 *	0.006	*0.461 *	0.008	*0.298 *
SBP			−0.001	*0.618 *	−0.002	*0.350 *	−0.002	*0.378 *	−0.003	*0.219 *
HbA1c			0.062	*0.180 *	0.057	*0.211 *	0.065	*0.157 *	0.057	*0.206 *
BMI			0.027	*0.030 *	0.063	*0.095 *	0.035	*0.007 *	0.157	*0.001 *
log-Leptin × BMI					−0.053	*0.015 *			−0.078	*0.011 *
log-Adipo × BMI					0.009	*0.796 *			−0.078	*0.076 *
log-Leptin × site							−0.272	*0.354 *	0.449	*0.279 *
log-Adipo × site							0.758	*0.018 *	1.288	*0.002 *

Coefficients and *P* values from multiple linear regression models used to examine relations of log-leptin, log-adiponectin to log-CRP for men are shown. PA index: physical activity index score; SBP: systolic blood pressure; HbA1c: hemoglobin A1c; BMI: body mass index; site (Hawaii = 1, Japan = 0).

**Table 5:** Relations of serum log-leptin and log-adiponectin to log-CRP in women.

	Model 1		Model 2		Model 3		Model 4		Model 5	
	*β*	*P *	*β*	*P *	*β*	*P *	*β*	*P *	*β*	*P *
Site	0.336	*<0.001 *	0.314	*<0.001 *	0.313	*<0.001 *	0.729	*0.166 *	0.871	*0.123 *
Age	0.016	*0.004 *	0.014	*0.018 *	0.014	*0.016 *	0.013	*0.026 *	0.013	*0.024 *
log-Leptin	0.636	*<0.001 *	0.268	*0.153 *	0.118	*0.880 *	0.324	*0.122 *	−0.071	*0.929 *
log-Adiponectin	−0.405	*0.016 *	−0.320	*0.059 *	−1.005	*0.364 *	−0.200	*0.370 *	−1.189	*0.290 *
Cigarettes/day			−0.008	*0.586 *	−0.007	*0.642 *	−0.008	*0.590 *	−0.006	*0.664 *
PA index			−0.012	*0.180 *	−0.012	*0.170 *	−0.012	*0.173 *	−0.012	*0.165 *
SBP			0.002	*0.408 *	0.002	*0.446 *	0.002	*0.428 *	0.002	*0.463 *
HbA1c			0.002	*0.976 *	0.001	*0.980 *	0.003	*0.965 *	0.002	*0.974 *
BMI			0.031	*0.004 *	−0.002	*0.982 *	0.032	*0.005 *	−0.028	*0.712 *
log-Leptin × BMI					0.006	*0.858 *			0.017	*0.623 *
log-Adipo × BMI					0.029	*0.530 *			0.043	*0.369 *
log-Leptin × site							−0.150	*0.629 *	−0.206	*0.530 *
log-Adipo × site							−0.273	*0.411 *	−0.362	*0.302 *

Coefficients and *P* values from multiple linear regression models used to examine relations of log-leptin, log-adiponectin to log-CRP for men are shown. PA index: physical activity index score; SBP: systolic blood pressure; HbA1c: hemoglobin A1c; BMI: body mass index; site (Hawaii = 1, Japan = 0).
